# Black Oxide of Mercury in the Vomiting of Pregnancy

**Published:** 1847-02

**Authors:** 


*Use of Black Oxide of Mercury in the Vomiting of Pregnancy.*—M. Stackler has announced that he has found the black oxide of mercury a most useful medicine for removing the obstinate vomiting which sometimes occurs in females during pregnancy. The vomiting was checked in two cases of unusual severity, by doses of the oxide, equal to five centigrammes daily (=0·77 grains). No unpleasant effects were observed to follow the use of the medicine. There was no salivation. Doctor Jouger has found the oxide equally beneficial in hysteric convulsons and in cases of uterine irritation. *Ibid.—Southern Jour. Med. and Phar.*

